# Glycemic Outcomes in Adults With Type 2 Diabetes Participating in a Continuous Glucose Monitor–Driven Virtual Diabetes Clinic: Prospective Trial

**DOI:** 10.2196/21778

**Published:** 2020-08-28

**Authors:** Amit R Majithia, Coco M Kusiak, Amy Armento Lee, Francis R Colangelo, Robert J Romanelli, Scott Robertson, David P Miller, David M Erani, Jennifer E Layne, Ronald F Dixon, Howard Zisser

**Affiliations:** 1 Department of Medicine and Department of Pediatrics University of California San Diego School of Medicine La Jolla, CA United States; 2 Verily Life Sciences South San Francisco, CA United States; 3 Allegheny Health Network Monroeville, PA United States; 4 Sutter Health Palo Alto, CA United States; 5 Onduo Professionals, PC Newton, MA United States; 6 Onduo, LLC Newton, MA United States

**Keywords:** continuous glucose monitoring, telemedicine, telehealth, digital health, type 2 diabetes, HbA1c

## Abstract

**Background:**

The Onduo virtual diabetes clinic (VDC) for people with type 2 diabetes (T2D) combines a mobile app, remote personalized lifestyle coaching, connected devices, and live video consultations with board-certified endocrinologists for medication management and prescription of real-time continuous glucose monitoring (RT-CGM) devices for intermittent use.

**Objective:**

This prospective single-arm study evaluated glycemic outcomes associated with participation in the Onduo VDC for 4 months.

**Methods:**

Adults aged ≥18 years with T2D and a baseline glycated hemoglobin (HbA1c) of ≥8% to ≤12% were enrolled from 2 primary care centers from February 2019 to October 2019. Participants were asked to engage at ≥1 time per week with their care team and to participate in a telemedicine consultation with a clinic endocrinologist for diabetes medication review. Participants were asked to use a RT-CGM device and wear six 10-day sensors (total 60 days of sensor wear) intermittently over the course of 4 months. The primary outcome was change in HbA1c at 4 months from baseline. Other endpoints included change in weight and in RT-CGM glycemic metrics, including percent time <70, 70-180, 181-250, and >250 mg/dL. Changes in blood pressure and serum lipids at 4 months were also evaluated.

**Results:**

Participants (n=55) were 57.3 (SD 11.6) years of age, body mass index 33.7 (SD 7.2), and 40% (22/55) female. HbA1c decreased significantly by 1.6% (SD 1%; *P*<.001). When stratified by baseline HbA1c of 8.0% to 9.0% (n=36) and >9.0% (n=19), HbA1c decreased by 1.2% (SD 0.6%; *P*<.001) and 2.4% (SD 1.3%; *P*<.001), respectively. Continuous glucose monitoring–measured (n=43) percent time in range (TIR) 70-180 mg/dL increased by 10.2% (SD 20.5%; *P*=.002), from 65.4% (SD 23.2%) to 75.5% (SD 22.7%), which was equivalent to a mean increase of 2.4 hours TIR per day. Percent time 181-250 mg/dL and >250 mg/dL decreased by 7.2% (SD 15.4; *P*=.005) and 3.0% (SD 9.4; *P*=.01), respectively. There was no change in percent time <70 mg/dL. Mean weight decreased by 9.0 lb (SD 10.4; *P*<.001). Significant improvements were also observed in systolic blood pressure, total cholesterol, low-density lipoprotein cholesterol, and triglycerides (*P*=.04 to P=<.001).

**Conclusions:**

Participants in the Onduo VDC experienced significant improvement in HbA1c, increased TIR, decreased time in hyperglycemia, and no increase in hypoglycemia at 4 months. Improvements in other metabolic health parameters including weight and blood pressure were also observed. In conclusion, the Onduo VDC has potential to support people with T2D and their clinicians between office visits by increasing access to specialty care and advanced diabetes technology including RT-CGM.

**Trial Registration:**

ClinicalTrials.gov NCT03865381; https://clinicaltrials.gov/ct2/show/NCT03865381

## Introduction

In recent years, there has been a dramatic increase in telehealth programs for the management of diabetes [1]. These programs have the potential to support about 34 million individuals with diabetes in the United States and may play a larger role in future diabetes care, given the expected increase in the incidence and prevalence of diabetes in the next decade and beyond [2]. Telehealth programs for diabetes typically include a smartphone app, connected devices such as blood glucose meters, and remote coaching that may be automated or provided by a live health coach. Current telehealth programs, however, do not address the limitations of the traditional health care model of diabetes management: limited access to endocrinologists, specialist education, and advanced diabetes management technology.

There is growing recognition that advanced technology, including continuous glucose monitoring (CGM) devices, can play an essential role in diabetes care for people with type 2 diabetes (T2D), regardless of their treatment regimen [3-7]. The Onduo Virtual Diabetes Clinic (VDC), a telehealth model for people with T2D, is unique in incorporating CGM in its care model. Availability of live video consultations with board-certified endocrinologists for medication management and the ability to remotely prescribe CGM devices are also unique components of the VDC. Real-world evidence suggests that participation in the VDC is associated with significant improvement in HbA_1c_ [8] and a significant reduction in diabetes distress [9]. Here we report outcomes of a prospective single-group assignment trial, examining changes in HbA_1c_ in adults with T2D after 4 months of participation in the Onduo VDC.

## Methods

### Study Objective

The primary objective of this prospective single-arm study was to evaluate the change in HbA_1c_ in adults with T2D after 4 months of participation in the Onduo VDC. Additional outcomes included change in glycemic metrics from CGM (mean glucose, coefficient of variation, and percent time <70 mg/dL, 70 to 180 mg/dL [time in range (TIR)], 181 to 250 mg/dL, and >250 mg/dL at 4 months from baseline.Changes in weight, blood pressure (BP), and serum lipids—namely, total cholesterol, high-density lipoprotein (HDL) cholesterol, triglycerides, low-density lipoprotein (LDL) cholesterol, cholesterol/HDL ratio, and non-HDL cholesterol—were also evaluated at 4 months from baseline.

### Participants

Participants were enrolled from 2 primary care networks—Allegheny Health Network, Pittsburgh, PA and Sutter Health Palo Alto Medical Foundation, Palo Alto, CA. Inclusion criteria were as follows: ≥18 years of age or older, confirmed diagnosis of T2D, HbA_1c_ level 8.0% and 12.0%, willingness to use a blood glucose meter and CGM device, and own a smartphone. Exclusion criteria were as follows: use of an insulin pump; pregnant or breastfeeding; malignant cancer in the previous 12 months; any solid organ transplant; end-stage (stage 4 or 5) renal disease or dialysis; liver failure; cystic fibrosis; chronic heart failure (Class C, D); diabetes-related pancreatic failure; current use of a blood thinner; and self-reported adhesive allergy. All participants provided written informed consent. The study protocol and consent forms were approved by the Western Institutional Review Board and registered with ClinicalTrials.gov NCT03865381.

### Protocol

Baseline and final assessments were conducted in-person at the designated study sites including, physical measures, blood draws, and questionnaires. The intervention was conducted remotely through the Onduo VDC.

### Virtual Diabetes Clinic Participation

The Onduo VDC telehealth program for people with T2D has been previously described [8,9]. In brief, the program combines mobile app technology, remote personalized lifestyle coaching from Certified Diabetes Care and Education Specialists (CDCES) and health coaches, and connected blood glucose meters and real-time continuous glucose monitoring (RT-CGM) devices. Live video consultations with board-certified endocrinologists are available as needed for medication management in addition to prescribing CGM. Participants interact with their care team and are sent educational materials by messaging through the app. Participants use the app to track data relevant to diabetes care (such as, blood glucose readings and CGM data) and to log medication use, physical activity, meal photos, and other information.

In this study, participants were asked to engage ≥1 time per week with their health coach or care team and to participate in a telemedicine consultation with VDC endocrinologists. All participants were mailed a RT-CGM device—Dexcom G6 (Dexcom)—for intermittent use. Participants were asked to wear six 10-day sensors (total 60 days of sensor wear) intermittently over the course of 4 months. Initial period of CGM device–wearing lasted for 20 days (consisting of 2 sensors worn back to back). Subsequently, the remaining 4 sensors were deployed in a 10-days “on” and 11-days “off” cycle. Sensor glucose data were used by the care team for coaching and monitoring and as an educational feedback loop to assist participants in associating their glucose levels with their diet, lifestyle, and other factors to optimize diabetes self-management. Glucose data were also used by the VDC endocrinologists for medication management. During the 4-month period, additional CGM sensor wear may have been requested by VDC endocrinologists to evaluate the efficacy of medication changes and monitor impact on blood glucose.

### Statistical Analysis

A sample size of 60 participants was selected to provide 90% power to detect a 0.5% decrease in HbA_1c_ at 4 months from baseline (primary outcome) using a 2-sided test with α=.05, after assuming 20% loss to follow-up. RT-CGM glycemic metrics, including mean glucose, coefficient of variation, and percent time <70 mg/dL, 70 to 180 mg/dL, 181 to 250 mg/dL, and >250 mg/dL) were calculated from an initial 10-day period within 30 days of enrollment to a 10-day follow-up period >90 days from enrollment, with a data sufficiency requirement of >70% of possible readings. All outcomes were evaluated by paired *t* test except for ranges <70 mg/dL and >250 mg/dL, which were evaluated by Wilcoxon signed rank test. Nominal significance levels (*P* values) are presented with statistical significance defined as *P*<.05. All statistical analyses were performed using Python 3.6.7.

## Results

A total of 60 participants enrolled in the study, and 92% (55/60) completed the 4-month intervention. Reasons for withdrawal included protocol violations/non-compliance (n=3) and withdrawal of consent (n=2). Out of 55 participants who completed the study, 89% (49/55) had a medication change. Baseline demographic and clinical characteristics of the participants are presented in Table 1.

**Table 1:** Participant demographics at baseline.

Variables		Values
Female, n (%)		22 (40)
Age (years) mean (SD)		57.3 (11.6)
Weight (lb) mean (SD)		218.7 (59.7)
BMI,^a^ mean (SD)		33.7 (7.2)
Baseline HbA_1c_, (%) mean (SD)		8.9 (1.0)
Systolic blood pressure (mm Hg) mean (SD)		132.1 (15.8)
Diastolic blood pressure (mm Hg) mean (SD)		80.7 (10.4)
Total cholesterol (mg/dL) mean (SD)		168.3 (42.8)
HDL^b^ cholesterol (mg/dL) mean (SD)		40.4 (9.1)
LDL^c^ cholesterol (mg/dL) mean (SD)		100.1 (36.5)
Non-HDL cholesterol (mg/dL) mean (SD)		128.0 (42.70)
Total cholesterol/HDL ratio, mean (SD)		4.4 (1.4)
Triglycerides (mg/dL) mean (SD)		236.7 (194.3)
**Diabetes medications, n (%)**		
	0	0 (0)
	1	6 (11)
	2	21 (38)
	≥3	28 (51)
**Type of diabetes medications, n (%)**		
	Alpha glucosidase inhibitor	1 (2)
	Biguanide	46 (84)
	DPP-4^d^ inhibitor	10 (18)
	GLP-1^e^ analogue	14 (25)
	Insulin	20 (36)
	SGLT2^f^ inhibitor	20 (36)
	Sulfonylurea	30 (55)
	Thiazolidinedione	2 (4)
	Lipid-lowering medications	44 (80)

^a^BMI: body mass index.

^b^HDL: high-density lipoprotein.

^c^LDL: low-density lipoprotein.

^d^DPP-4: dipeptidyl peptidase-4.

^e^GLP-1: glucagon-like peptide-1.

^f^SGLT2: sodium/glucose cotransporter 2.

### Change in HbA1c

**Figure 1:**
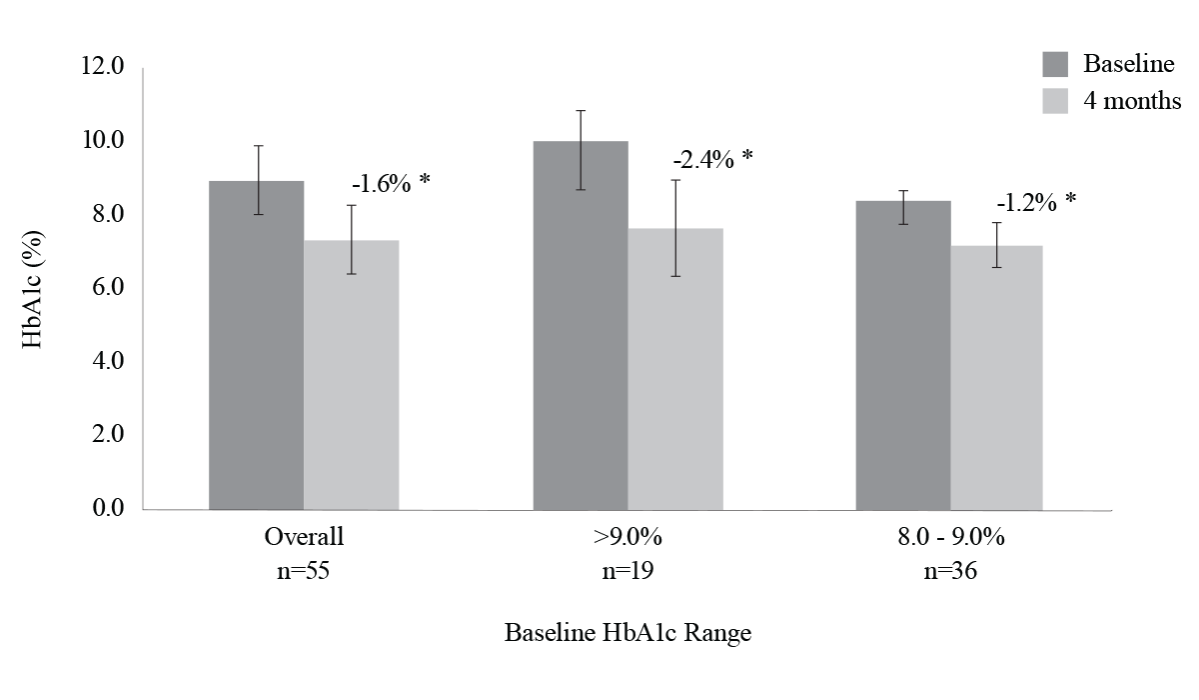
Change in HbA1c at 4 months from baseline. *P<.001.

HbA_1c_ decreased significantly by 1.6% (SD 1%; *P*<.001), from 8.9% (SD 1%) at baseline to 7.3% (SD 0.9%) at 4 months **(**Figure 1**)**. When stratified by baseline HbA_1c_ of 8.0% to 9.0% (n=36) and >9.0% (n=19), HbA_1c_ decreased by 1.2% (SD 0.6%) and 2.4% (SD 1.3%), respectively (both *P*<.001).

### CGM Device Use

Out of 55 participants in the study, 78.2% (43/55) met the criteria of follow-up CGM sensor–wear periods >90 days from baseline. Sensor-wear period was 94.8% (SD 8.2%) of the time specified per protocol (60 days) in this cohort. CGM metrics (n=43) are presented in Table 2. The increase in percent time in range 70 to 180 mg/dL was equivalent to a mean increase of 2.4 hours TIR per day.

**Table 2:** Changes in CGM metrics at 4 months from baseline.

Parameter		Baseline Mean (SD)	Follow-up Mean (SD)	Change Mean (SD)	*P* value
Mean glucose (mg/dL)		169.2 (29.3)	154.6 (33.0)	–14.6 (27.5)	.001
Coefficient of variation (%)		24.5 (4.9)	22.9 (5.5)	–1.6 (4.5)	.02
**Percent time (%)**					
	<70 mg/dL	0.2 (0.4)	0.3 (0.6)	0.1 (0.7)	.49
	70 to 180 mg/dL	65.4 (23.2)	75.5 (22.7)	10.2 (20.5)	.002
	181 to 250 mg/dL	26.5 (14.9)	19.2 (15.5)	–7.2 (15.4)	.005
	>250 mg/dL	8.0 (10.9)	5.0 (11.0)	–3.0 (9.4)	0.01

### Weight, BP, and Serum Lipids

Change in weight, BP, and serum lipids at 4 months from baseline are presented in Table 3. Significant decreases were observed in weight, body mass index (BMI), systolic BP, total cholesterol, LDL cholesterol, total cholesterol/HDL ratio and triglycerides (*P*=.04 to *P*<.001). 

**Table 3:** Change in BP and serum lipids at 4 months from baseline.

Parameter	Baseline Mean (SD)	Follow-up Mean (SD)	Change Mean (SD)	*P* value
Weight (lb)^a^	217.5 (59.5)	208.5 (53.7)	–9.0 (10.4)	<.001
BMI^a^^b^	33.6 (7.2)	32.2 (6.5)	–1.34 (1.5)	<.001
Systolic BP^a^^c^ (mm Hg)	132.4 (15.8)	128.0 (16.6)	–4.4 (13.1)	.04
Diastolic BP (mm Hg)^a^	80.5 (10.5)	79.8 (10.5)	–0.8 (7.5)	0.48
Total cholesterol (mg/dL)	168.3 (42.8)	151.7 (41.1)	–16.6 (46.0)	<.001
HDL^a^^d^ cholesterol, (mg/dL)	40.4 (9.1)	40.0 (10.9)	–0.4 (7.1)	.90
LDL^a^^e^ cholesterol (mg/dL)	100.1 (36.5)	93.6 (31.9)	–6.5 (27.5)	.04
Total cholesterol/HDL Ratio	4.4 (1.4)	3.9 (1.3)	–0.5 (1.4)	.003
Triglycerides (mg/dL)	236.7 (194.30)	193.0 (163.2)	–43.7 (115.4)	.008

^a^For n=54 (1 subject did not complete the 4-month assessment at the study site, but submitted results from an external laboratory).

^b^BMI: body mass index.

^c^BP: blood pressure.

^d^HDL: high-density lipoprotein.

^e^LDL: low-density lipoprotein.

### Adverse Events

There were no serious adverse events.

## Discussion

In this prospective single-arm trial of the Onduo VDC, adults with T2D and suboptimal glycemic control experienced a statistically significant and clinically meaningful reduction in HbA_1c_ at 4 months. Analysis of RT-CGM metrics demonstrated a significant increase in TIR, decreased time in hyperglycemia, and found no increase in hypoglycemia. Participants also experienced significant decreases in weight, systolic BP, and serum lipids. Delivering RT-CGM devices, incorporating insights from intermittent RT-CGM use, and evaluating glycemic outcomes using RT-CGM data are unique aspects of the overall Onduo VDC care model for people with T2D.

Erhardt et al [4] and Vigersky et al [5] have reported on the use of intermittent RT-CGM in a population with T2D in a 52-week, two-arm, randomized controlled trial (RCT) that compared a 12-week active intervention of intermittent RT-CGM use to self-monitoring blood glucose. Similar to the present study, this RCT [4,5] also utilized Dexcom RT-CGM devices on an intermittent basis (2 weeks use, 1 week off) and measured short-term change in HbA_1c_. Participants in the RCT (RT-CGM group n=50) and the present study were predominately male, similar for age, baseline HbA_1c_ and insulin use. Health insurance coverage differed: RCT participants were military health care beneficiaries compared to a mainly commercially insured population in the present study. In addition, the present study integrated intermittent RT-CGM data in Onduo VDC’s coaching and telemedicine care model, while limited information was reported regarding the use of RT-CGM data in the RCT. A mean decrease in HbA_1c_ of 1% was observed in the RCT RT-CGM group vs 1.6% in the present study, suggesting a potential additive or synergistic benefit of combining intermittent RT-CGM and telehealth in people with T2D. Interestingly, TIR at the final assessment was similar in the RCT intervention group and the present study, 75.3% and 75.5%, respectively. During the 40-week follow-up period, RCT participants did not use RT-CGM, yet durable improvement in HbA_1c_ was observed at the 52-week follow-up assessment in the RT-CGM group compared with the self-monitoring blood glucose group. Similarly, the duration of our intervention was 4 months, and an evaluation of outcomes at 12-month outcomes is planned.

Previous studies of telehealth interventions in individuals with T2D have reported reductions in HbA_1c_ ranging from 0.7% to 2.1% [10-14]. Some of these studies have also reported improvements in secondary outcomes such as weight, lipids, and BP, although results are variable [11,13-16]. Direct comparisons to prior telehealth studies are complicated by differences in participant demographics, length of intervention, program features, and method of reporting HbA_1c_, for example, directly measured vs estimated from fingerstick blood glucose readings. The magnitude of HbA_1c_ reduction in this prospective trial is consistent with that observed at an average of 4 months in a recently published retrospective analysis of 740 VDC participants [8]. Specifically, in participants with a baseline HbA_1c_ ≥8%, HbA_1c_ declined by 1.5% in the retrospective study and by 1.6% in the present study. In participants with baseline HbA_1c_ >9.0%, HbA_1c_ declined by 2.3% in the retrospective study and 2.4%, in this study.

Importantly, this is the first telehealth study in which participants with T2D were provided RT-CGM devices and changes in RT-CGM metrics beyond HbA_1c_ were quantified. Providing RT-CGM to participants in the VDC program supports individualized diabetes management in 2 ways. First, it improves self-management by showing participants how specific diet and lifestyle choices impact their blood glucose fluctuations. Second, rich information on glycemic variability that is best derived from CGM data provides information to VDC endocrinologists, pharmacists, nutritionists, CDCES, and health coaches to guide lifestyle and therapeutic interventions.

At a population level, assessing changes in RT-CGM–derived glycemic outcomes supplements HbA_1c_, offering additional insight into the impact of an intervention on diabetes management. Notably, the mean 10% increase in TIR observed in this study—equivalent to an increase of 2.4 hours per day—is considered a clinically meaningful increase [17]. Increased TIR is associated with lower vascular complication burden [5]. People with T2D report that TIR has at least as significant impact as HbA_1c_ has on their daily life [18]. The dual application of RT-CGM as a therapeutic and diagnostic tool is a unique strength of the Onduo VDC program and this study.

Limitations of our study include the sample size, duration of the intervention, and lack of a randomized control arm. It is important to note that some of the observed decrease in HbA_1c_ may be attributed to regression to the mean. Further studies are planned.

This prospective clinical trial of the Onduo VDC demonstrated improvements in glycemic outcomes in adults with suboptimally controlled T2D. Improvements in risk factors for diabetes complications, including weight, BP, and serum lipids were also observed.

## Abbreviations

BMI: body mass index
BP: blood pressure
CDCES: Certified Diabetes Care and Education Specialist
CGM: continuous glucose monitoring
HbA1c: glycated hemoglobin
HDL: high-density lipoprotein
LDL: low-density lipoprotein
RCT: randomized controlled trial
RT-CGM: real-time continuous glucose monitoring
T2D: type 2 diabetes
TIR: time in range
VDC: virtual diabetes clinic
